# Hemoadsorption therapy in the critically ill: solid base but clinical haze

**DOI:** 10.1186/s13613-019-0491-1

**Published:** 2019-01-31

**Authors:** Patrick M. Honoré, David De Bels, Leonel Barreto Gutierrez, Herbert D. Spapen

**Affiliations:** 1 ICU Department, Centre Hospitalier Universitaire Brugmann, Place Van Gehuchtenplein, 4, 1020 Brussels, Belgium; 2 Ageing and Pathology Research Group, Vrije Universiteit Brussel, Brussels, Belgium

In a recent issue of Annals of Intensive Care, Xu et al. [1] studied the efficacy of HA330 resin-directed hemoadsorption (HA) in an endotoxin-induced porcine acute respiratory distress syndrome (ARDS) model. HA330-HA improved oxygenation and lung mechanics, blunted lung edema and histopathological signs of ARDS, reduced circulating and alveolar cytokine levels, and profoundly changed plasma and lung proteome. The authors hypothesized that HA330-HA could beneficially influence the course of ARDS by attenuating systemic and pulmonary inflammatory cytokine “overshooting” and by restoring disordered proteome homeostasis in the exudative phase [1].

This meticulously executed and extensively documented study puts adjuvant sorbent-based treatment in acute severe inflammatory disease in bright spotlight. HA is an extracorporeal technique involving the passage of blood or plasma through a cartridge where solutes are removed by direct binding to the sorbent material. Cartridges substantially differ from hemofilters. A hemofilter consists of thousands of tiny hollow fibers which structurally mimic the nephron, while a cartridge contains adsorbing beads covering a surface area that by far exceeds that of a hemofilter [2]. Cartridges are divided in selective (e.g., polymyxin B hemoperfusion (PMX-HP)) and non-selective types (e.g., CytoSorb^®^) [3]. Cartridge selectivity may have important consequences for treatment. For instance, non-selective cartridges cannot adsorb endotoxin because their cutoff point (~ 60 kDa) falls below the molecular weight of endotoxin (~ 100 kDa) [2, 3]. In contrast, PMX-HP selectively adsorbs endotoxin even when up to 95% of the endotoxin in the body is lipid-bound [4].

The Jafron HA resin hemoperfusion cartridges belong to the non-selective group. Different types of cartridges (HA-130, HA-230, HA-330) have been developed. The difference in pore size distribution makes them applicable in settings varying from reduction of uremic symptoms in chronic hemodialysis (HA-130) and treatment of paraquat and organophosphorus poisoning (HA-230) to modulation of severe inflammatory processes (HA-330) [5]. The data provided by Xu et al. [1] corroborate the results of two small randomized controlled trials (RCT) investigating adjuvant HA330-HA in septic patients with acute lung injury. Compared with controls, HA330-HA-treated patients had a significantly less inflammatory cytokine “load” in plasma and lung tissue. This was associated with improved hemodynamic and respiratory parameters, lower intensive care unit (ICU) length of stay, reduced ICU mortality, and no safety concerns [6, 7]. However, these studies were largely underpowered.

Of note is that clinical experience with the Jafron HA cartridges is mainly limited to China. A counterpart of HA330 is the equally non-selective extracorporeal cytokine adsorber CytoSorb^®^. The CytoSorb^®^ device became clinically available in 2011 and is currently the only approved extracorporeal adsorption technique in the European Union. CytoSorb^®^ is essentially indicated to control a detrimental cytokine storm in critically ill and cardiac surgery patients but can be used in all non-infectious conditions characterized by systemic hyperinflammation (e.g., polytrauma, burns, trauma, pancreatitis). Data from an international registry, including 68% patients with sepsis, showed that CytoSorb^®^ therapy markedly reduced interleukin (IL)-6 levels. No significant decrease in organ failure was observed, but hospital mortality was lower than predicted [8]. A multicenter RCT evaluated CytoSorb^®^ treatment in 97 patients with severe sepsis or septic shock and acute lung injury or ARDS [9]. The CytoSorb^®^ cartridge was either used alone or inserted proximally into a conventional continuous veno-venous hemofiltration/hemodiafiltration circuit. Compared with controls, CytoSorb^®^ HA failed to lower plasma IL-6 levels (primary endpoint) and caused no significant differences in incidence of organ failure, respiratory variables, duration of ventilation, and adjusted mortality [9]. This negative study, however, has been criticized on methodological grounds. A recent proof of concept, prospective, randomized pilot trial investigating the effects of early (< 24 h) stand-alone CytoSorb^®^ treatment in 20 patients with septic shock reported improved hemodynamics and significantly lower levels of procalcitonin and an endothelin-1 precursor [10]. This study underscores the importance of defining appropriate clinical and biological endpoints when assessing HA therapy.

Within this context, Xu et al. have added highly valuable experimental evidence supporting the benefits of an HA strategy in a critical inflammatory setting. So far, strong and straightforward data sustaining the clinical implementation of this approach are lacking. Large prospective trials in carefully selected patient populations and well-defined conditions are needed to definitely evaluate the efficacy of sorbent devices.

## Abbreviations

HA: hemoadsorption
ARDS: acute respiratory distress syndrome
PMX-HP: polymyxin B hemoperfusion
RCT: randomized controlled trial
ICU: intensive care unit
IL: interleukin

## References

[1] Xu X, Jia C, Luo S, Li Y, Xiao F, Dai H (2017). Effect of HA330 resin-directed hemoadsorption on a porcine acute respiratory distress syndrome model. Ann Intensive Care.

[2] Honoré PM, De Bels D, Spapen HD (2018). An update on membranes and cartridges for extracorporeal blood purification in sepsis and septic shock. Curr Opin Crit Care.

[3] Honore PM, Jacobs R, Joannes-Boyau O, De Regt J, De Waele E, van Gorp V (2013). Newly designed CRRT membranes for sepsis and SIRS—a pragmatic approach for bedside intensivists summarizing the more recent advances: a systematic structured review. ASAIO J.

[4] Ronco C, Klein DJ (2014). Polymyxin B hemoperfusion: a mechanistic perspective. Crit Care.

[5] Ankawi G, Fan W, Pomarè Montin D, Lorenzin A, Neri M, Caprara C (2018). A new series of sorbent devices for multiple clinical purposes: current evidence and future directions. Blood Purif.

[6] Huang Z, Wang SR, Su W, Liu JY (2010). Removal of humoral mediators and the effect on the survival of septic patients by hemoperfusion with neutral microporous resin column. Ther Apher Dial.

[7] Huang Z, Wang SR, Yang ZL, Liu JY (2013). Effect on extrapulmonary sepsis-induced acute lung injury by hemoperfusion with neutral microporous resin column. Ther Apher Dial.

[8] Friesecke S, Träger K, Schittek GA, Molnar Z, Bach F, Kogelmann K (2017). International registry on the use of the CytoSorb^®^ adsorber in ICU patients: study protocol and preliminary results. Med Klin Intensivmed Notfmed.

[9] Schädler D, Pausch C, Heise D, Meier-Hellmann A, Brederlau J, Weiler N (2017). The effect of a novel extracorporeal cytokine hemoadsorption device on IL-6 elimination in septic patients: a randomized controlled trial. PLoS ONE.

[10] Hawchar F, László I, Öveges N, Trásy D, Ondrik Z, Molnar Z (2019). Extracorporeal cytokine adsorption in septic shock: a proof of concept randomized, controlled pilot study. J Crit Care.

